# Factores que afectan la cobertura del programa de tuberculosis en el primer nivel de atención en Honduras

**DOI:** 10.7705/biomedica.6368

**Published:** 2022-06-01

**Authors:** Briana Beltrán, Dione Benjumea-Bedoya, Jackeline Alger

**Affiliations:** 1 Grupo de Epidemiología, Facultad Nacional de Salud Pública, Universidad de Antioquia, Medellín, Colombia Universidad de Antioquia Grupo de Epidemiología Facultad Nacional de Salud Pública Universidad de Antioquia Medellín Colombia; 2 Centro Nacional de Educación Médica Continua, Colegio Médico de Honduras, Tegucigalpa, Honduras Centro Nacional de Educación Médica Continua Colegio Médico de Honduras Tegucigalpa Honduras; 3 Grupo de Investigación en Salud Familiar y Comunitaria, Facultad de Ciencias de la Salud, Corporación Universitaria Remington, Medellín, Colombia Corporación Universitaria Remington Grupo de Investigación en Salud Familiar y Comunitaria Facultad de Ciencias de la Salud Corporación Universitaria Remington Medellín Colombia; 4 Unidad de Investigación Científica, Facultad de Ciencias Médicas, Universidad Nacional Autónoma de Honduras, Tegucigalpa, Honduras Unidad de Investigación Científica Facultad de Ciencias Médicas Universidad Nacional Autónoma de Honduras Tegucigalpa Honduras; 5 Departamento de Laboratorio Clínico, Hospital Escuela, Tegucigalpa, Honduras Departamento de Laboratorio Clínico Hospital Escuela, Tegucigalpa Honduras

**Keywords:** tuberculosis, accesibilidad a los servicios de salud, actitud del personal de salud, barreras de acceso a los servicios de salud, tuberculosis pulmonar, Honduras, Tuberculosis, health services accessibility, attitude of health personnel, barriers to access to health services, tuberculosis, pulmonary, Honduras

## Abstract

**Introducción.:**

Hay consenso global en que el diagnóstico y el tratamiento precoces de la tuberculosis pueden acelerar su control y mitigar sus consecuencias. En Honduras, la tasa de mortalidad por la enfermedad aumentó gradualmente entre 2014 y 2018, a lo que se suman las reformas en el sistema de salud del 2014 y la implementación parcial de la estrategia “Fin a la TB”.

**Objetivo.:**

Analizar las barreras y los elementos facilitadores del diagnóstico y el tratamiento que afectan la cobertura del programa nacional de tuberculosis, con el fin de brindar herramientas para la implementación efectiva de la estrategia “Fin a la TB” en San Pedro Sula, Honduras, 2015-2019.

**Materiales y métodos.:**

Se hizo un estudio mixto secuencial y explicativo de pacientes mayores de 18 años con tuberculosis pulmonar positivos en la baciloscopia. Se revisaron las fichas de notificación de la enfermedad y las historias clínicas en dos establecimientos de salud de primer nivel y se hicieron entrevistas semiestructuradas al personal de salud, los pacientes y los familiares.

**Resultados.:**

En el 74,6 % (297/398) de los casos no hubo diagnóstico oportuno. En este grupo, se encontró una mayor proporción de hombres (62,3 %; 185/297) y de adultos (80,8 %; 240/297); predominó un nivel de escolaridad inferior a la secundaria (53,7 %; 108/297); el 49,2 % (123/297) de los pacientes tenía alguna ocupación, y el 98,2 % había recibido tratamiento oportuno. Se detectaron las siguientes barreras: condiciones socioeconómicas precarias, desarticulación del sistema de salud público y privado, y límites fronterizos entre maras y pandillas. Los elementos facilitadores fueron la buena atención y la actitud del personal de salud, y la disponibilidad y reserva de tratamiento.

**Conclusiones.:**

La falta de oportunidad en el diagnóstico de tuberculosis afectó la cobertura del programa nacional como resultado de las barreras culturales y de atención en salud.

## Abstract (en)

**Introduction:**

There is a global consensus that early diagnosis and treatment of tuberculosis (TB) can accelerate its control and mitigate its consequences. The gradual increase in the TB mortality rate from 2014 to 2018 in Honduras, the reform of the health system in 2014, and the partial implementation of the “End TB” strategy motivated this study.

**Objective:**

To analyze barriers to and facilitators of diagnosis and treatment affecting the national TB program coverage using data from 2015 to 2019 and provide tools for the effective implementation of the “End TB” strategy in San Pedro Sula, Honduras.

**Materials and methods:**

This was an explanatory sequential mixed-methods study on smear-positive pulmonary TB patients older than 18 years of age. TB notification sheets and medical records from two primary health care facilities were reviewed. Semistructured interviews were conducted with health care providers, patients, and their families.

**Results:**

A total of 74.6% of the cases (297/398) did not receive a timely diagnosis; 62.3% (185/297) were men, 80.8% (240/297) were adults, 53.7% (108/297) had less than high school education, 49.2% (123/297) had some occupation, and 98.2% of participants received timely treatment. Identified barriers included low socioeconomic conditions, lack of coordination between public and private health systems, and boundaries set by gangs. Identified facilitators included good care and attitude of the health care personnel and the availability of medications.

**Conclusions:**

The lack of opportunity to diagnose the disease affected the coverage of the national TB program due to cultural and health care barriers.

En el 2019, enfermaron de tuberculosis 10 millones de personas y 1,4 millones murieron por esta causa, lo que equivale a una tasa de letalidad del 14 % ^1^. Aunque desde el 2000 el tratamiento de la enfermedad ha evitado más de 60 millones muertes, la falta de cobertura universal sigue ocasionando brechas en el diagnóstico y la atención ^1^.

Según los reportes globales de la Organización Mundial de la Salud (OMS), entre el 2014 y el 2017 hubo en Honduras un incremento progresivo de la mortalidad por tuberculosis, la cual pasó de 1,5 % a 1,9 %, 4,8 % y 5,04 % en esos años ^2^^-^^6^. En el 2018, el país reportó 2.866 casos de la enfermedad en todas las formas ^7^.

Tras el acuerdo ejecutivo 406-2014 ^8^, se introdujeron cambios en la Secretaría de Salud de Honduras (SESAL), lo que ocasionó que desaparecieran los niveles centrales de, entre otros, los programas nacionales de tuberculosis y del síndrome de inmunodeficiencia adquirida (sida), y pasaran a ser dirigidos por otros departamentos o dependencias de la SESAL ^9^ en un esquema de horizontalidad. Con apoyo de la Organización Panamericana de la Salud (OPS), en el 2017 se encontró que las regiones de la Costa Atlántica requerían mayores esfuerzos para eliminar la tuberculosis ^10^; en ese contexto, se seleccionó la Región Sanitaria Metropolitana de San Pedro Sula, ubicada en el departamento de Cortés, para el presente estudio bajo un enfoque de investigación de la implementación, lo que permite examinar estrategias específicamente diseñadas para mejorar la ejecución de intervenciones de salud ^11^. En Honduras, no hay estudios recientes sobre tuberculosis con este enfoque de investigación, por lo que el objetivo general de nuestro estudio fue analizar las barreras y elementos facilitadores del diagnóstico y el tratamiento que afectan la cobertura del Programa Nacional de Tuberculosis, con el fin de aportar herramientas para la implementación efectiva de la estrategia “Fin a la TB” en San Pedro Sula, Honduras.

## Materiales y métodos

### Diseño

Se hizo un estudio mixto secuencial y explicativo en dos fases, una cuantitativa y otra cualitativa.

### Población

La población de la fase cuantitativa fueron pacientes mayores de 18 años con tuberculosis pulmonar y baciloscopia positiva. En la fase cualitativa se incluyó al personal de salud, a pacientes con diagnóstico de tuberculosis pulmonar y baciloscopia positiva, y a sus respectivos familiares.

### Contexto

El estudio se llevó a cabo en la Región Sanitaria Metropolitana de San Pedro Sula, departamento de Cortés, en dos establecimientos del primer nivel de atención: un policlínico y una unidad de atención primaria en salud.

### Variables

En la fase cuantitativa, las variables dependientes fueron: el diagnóstico oportuno, definido como “tiempo menor o igual a 30 días desde el inicio de los síntomas hasta la confirmación del hallazgo de *Mycobacterium tuberculosis* en la muestra de esputo ^12^; el tratamiento oportuno, definido como “la administración de la primera dosis de medicamento anti-TB en un tiempo menor o igual a tres días desde la confirmación de *Mycobacterium tuberculosis* en la muestra biológica” ^12^, y el número de días desde la indicación de la baciloscopia hasta el inicio del tratamiento. Las variables independientes incluyeron las características sociodemográficas y clínicas. La variable de la edad se ajustó en dos grupos: el de adultos, subdividido en adultos jóvenes (18-35 años) y adultos medios (36-64 años), y el de la tercera edad, es decir, los mayores de 65 años ^13^.

### Fuentes de datos

En la fase cuantitativa, se utilizaron fuentes secundarias de información: la ficha de casos de tuberculosis, las historias clínicas y el libro de cohorte de pacientes con la enfermedad; los datos se extrajeron utilizando un formato de verificación. En la fase cualitativa, se entrevistó al personal de salud, a los pacientes y a sus familiares mediante entrevistas semiestructuradas con preguntas revisadas por jueces expertos. En las dos fases se realizaron pruebas piloto.

### Tamaño de la muestra y estrategia de muestreo

En la fase cuantitativa, se utilizó el censo del periodo entre el 01-12-2015 y el 31-11-2019, y los siguientes criterios de elegibilidad: paciente mayor de 18 años con tuberculosis pulmonar, baciloscopia positiva y tratamiento registrado en la SESAL en el periodo de 2015 a 2019 en cualquiera de las dos unidades de salud de primer nivel de atención seleccionadas. En la fase cualitativa, el muestreo fue *a priori*^14^. La muestra del personal de salud incluyó a personas clave del Programa Nacional de Tuberculosis. Para facilitar su ubicación, los pacientes se seleccionaron solamente entre aquellos registrados de enero a junio del 2019 y se consideraron los siguientes criterios:

1. diagnóstico y tratamiento oportuno,
2. diagnóstico y tratamiento tardío,
3. finalización del tratamiento y
4. abandono del tratamiento.

Los familiares de los pacientes se escogieron entre quienes acompañaron al paciente durante la enfermedad. Cada entrevista duraba 20 minutos aproximadamente. Se entrevistaron seis miembros del personal de salud relacionados con el Programa Nacional de Tuberculosis en diversas funciones y con características o vivencias asociadas con la problemática de estudio, así como ocho pacientes y sus respectivos familiares, uno por paciente.

### Sesgos

Los sesgos de información del instrumento de recolección se controlaron verificando la calidad de las fichas de los casos de tuberculosis y las historias clínicas, y confrontando la información con el libro de cohorte de la enfermedad, con el fin de establecer la coherencia de los datos provenientes de las diferentes fuentes como criterio de calidad. Los sesgos de observador se controlaron mediante la operacionalización y estandarización rigurosas de los formatos de registro de la base de datos. Para controlar los sesgos de selección, se incluyó la información de los periodos definidos y se trabajó con la totalidad de los registros. Además, se revisaron datos perdidos y extremos. En la fase cualitativa, se consideraron como criterios de calidad los propios de la investigación cualitativa: los datos debían ser creíbles, confiables, y transferibles, poder confirmarse y reflejarse ^15^.

### Análisis de los datos

En la fase cuantitativa, se elaboró una base de datos en Microsoft Access® y se hizo la doble digitación del 20 % de los instrumentos, así como el análisis univariado y el bivariado e inferencial mediante la prueba de normalidad de Shapiro France. Al considerar dos variables cualitativas, se aplicó la prueba de ji al cuadrado o el test de Fisher según correspondiera.

En cuanto a los factores sociodemográficos y clínicos de interés, constituyentes de las variables independientes y de la variable dependiente de diagnóstico oportuno o no de los pacientes con baciloscopia positiva, se calcularon las razones de prevalencia cruda y ajustada, y se hizo la regresión binomial.

Se ingresaron al modelo las variables significativas que cumplían con el criterio de Hosmer-Lemeshow (p≤0,25) y se hizo un análisis de regresión de Cox, considerando el tiempo transcurrido desde el inicio de los síntomas respiratorios hasta el diagnóstico de la enfermedad, previendo que al emplear esta variable cuantitativa no se perdiera información. Se tomaron en cuenta el cociente de riesgo (*Hazard Ratio*, HR), un intervalo de confianza del 95 %, el valor de p y el criterio de información de Akaike (AIC). La información se procesó y analizó con los programas R Studio® 3.6 y SPSS, versión 25.

En cuanto a la fase cualitativa, las entrevistas se grabaron en audio y se transcribieron de manera textual en Microsoft Word™; luego, se procesaron y codificaron con el programa Nvivo, versión 12, y se les asignó una codificación alfanumérica. El análisis de contenido propio se hizo mediante lectura, codificación (abierta, axial y selectiva), presentación, reducción e interpretación ^14^^,^^16^. Además, se tuvieron en cuenta las categorías previamente estructuradas y las emergentes. Las categorías espec**í**ficas se estructuraron en categorías más generales, siguiendo el modelo ecológico social propuesto por McLeroy que se basa en el marco conceptual de Bronfenbrenner y ha sido adaptado por diversos autores ^17^^-^^20^.

Los niveles de este modelo se adaptaron así:

- el intrapersonal, es decir, el conocimiento de la enfermedad, creencias, disposición de protocolos, características del paciente y las experiencias;
- el interpersonal, o sea, el apoyo y la consejería del personal de salud brindada a los pacientes;
- el sistema de salud en cuanto a la accesibilidad a los servicios de salud, la atención al paciente con respecto al diagnóstico y tratamiento, y el tipo de articulación entre el sistema de salud público y el privado;
- el social y comunitario en lo relativo a la relación entre la sociedad y el paciente, y
- el político-administrativo, es decir, las diferentes reformas políticas en el sector salud y el funcionamiento horizontal con relación al Programa Nacional de Tuberculosis.

Para seleccionar los participantes de la fase cualitativa, en la triangulación se conectaron las fases cuantitativa y cualitativa con los datos cuantitativos recopilados y, a continuación, se integraron los resultados cuantitativos y cualitativos y sus respectivas implicaciones en la discusión ^21^.

### Aspectos éticos

Se obtuvo el aval del Comité de Ética de la Investigación de la Facultad Nacional de Salud Pública de la Universidad de Antioquia (No 21030002- 00258-2019) y del Comité de Ética en Investigación Biomédica de la Facultad de Ciencias Médicas de la Universidad Autónoma de Honduras (No 00003070). Se contemplaron las normas contenidas en la Declaración de Helsinki, el Código de Núremberg, el informe Belmont y la Resolución 8430 de 1993 del Ministerio de Salud de Colombia, la cual clasifica este tipo de estudio como de riesgo mínimo ^22^.

## Resultados

Entre el 2015 y el 2019, se diagnosticaron y trataron 454 pacientes con tuberculosis pulmonar y baciloscopia positiva en la Región Sanitaria Metropolitana de San Pedro Sula. De estos, 351 (77,3 %) pertenecían a la institución 1, y 103 (22,7 %) a la institución 2.

El 62,3 % (283) de los participantes era de sexo masculino, y la mitad de los pacientes tenía 43 años o más (rango intercuartílico (RIC): 28-57 años). El grupo etario con el mayor número de casos fue el de 25 a 34 años, con 24,2 % (110 casos), y el 92,1 % (418 casos) pertenecía a la población mestiza.

En cuanto a la escolaridad, el 35,9 % (160 casos) había alcanzado un nivel entre la primaria completa y la secundaria incompleta, en tanto que casi una tercera parte de los casos (30,6 %, 139/454 casos) no tenía trabajo (cuadro 1).

**Cuadro 1:** Características sociodemográficas y clínicas de los pacientes con tuberculosis pulmonar con baciloscopia positiva en dos establecimientos de salud de primer nivel de atención, Honduras, 2015-2019

Características		Establecimientos de salud				Total	
		Institución 1		Institución 2				
		n=351	%	n=103	%	n=454	%
Sexo	Hombre	220	62,7	63	61,2	283	62,3
	Mujer	131	37,3	40	38,8	171	37,7
Grupos etarios (años)	18-24	53	15,1	17	16,5	70	15,4
	25-34	83	23,6	27	26,2	110	24,2
	35-44	49	14	16	15,5	65	14,3
	45-54	58	16,5	20	19,4	78	17,2
	55-64	61	17,4	15	14,6	76	16,7
	≥65	47	13,4	8	7,8	55	12,1
Tipo de población	Mestizo	328	93,4	90	89,1	418	92,1
	Otro tipo de población	10	2,8	6	5,9	16	3,5
Grado de escolaridad	Primaria incompleta	92	26,2	26	25,7	118	25,9
	Primaria completa - secundaria incompleta	122	34,7	38	37,6	160	35,2
	Secundaria completa - nivel superior	24	9,1	0	2,9	24	5,3
	Ninguno	12	3,4	0	0,0	12	2,6
Ocupación	Sin trabajo	113	32,2	26	25,2	139	30,6
	Otras ocupaciones	145	41,3	52	51,5	98	43,4
	Ama de casa	31	8,8	11	10,7	42	9,3
Condición del paciente	Nuevo	316	90	96	93,2	412	90,7
	Recaída	33	9,4	5	4,9	38	8,4
	Abandono/recuperado	2	0,6	2	1,9	4	0,9
Diabetes mellitus	No	237	67,5	63	61,2	300	66,2
	Sí	82	23,4	26	25,2	108	23,9
Hipertensión arterial	No	271	77,2	80	77,7	351	77,4
	Sí	32	9,1	8	7,8	40	8,9

En cuanto a las características clínicas de los pacientes, el 90,7 % (412/454) de los casos era nuevo, y el 23,9 % (108 casos) tenía diabetes mellitus como comorbilidad (cuadro 2). En todos los participantes con baciloscopia positiva, se emplearon otras ayudas diagnósticas.

**Cuadro 2:** Días desde el inicio de los síntomas respiratorios hasta el inicio del tratamiento de los pacientes con tuberculosis pulmonar y baciloscopia positiva, y sus características sociodemográficas en dos establecimientos de salud de primer nivel de atención, Honduras, 2015-2019

Características		n	Días desde el inicio de síntomas respiratorios hasta la confirmación del diagnóstico	p	Tamaño del efecto r o E^2^_R_	Días desde la indicación de la baciloscopia hasta el comienzo deL tratamiento	p	Tamaño del efecto r o E^2^_R_
			---------------------- Mediana (RIC) ‡			------------------- Mediana (RIC)		
Sexo	Hombre	145	61 (31-122)	0,373	-0,040ᵝ	4 (2-6)	0,827	-0,011
	Mujer	253	61 (30-120)			4 (2-6)		
Grupos etarios (años)	18-24	64	61 (31-120)	0,935	0,003 ^Ω^	5 (2-8)	0,001	0,049
	25-34	97	61 (31-92)			4 (2-5)		
	35-44	57	61 (31-92)			5 (3-8)		
	45-54	65	61 (31-121)			5 (3-6)		
	55-64	69	61 (30-123)			4 (2-6)		
	≥65	46	70 (30-153)			3,5 (1-6)		
Tipo de población	Mestizo	369	61 (31-122)	0,699	-0,019	4 (2-6)	0,033	-0,108
	Otro tipo de población	14	76 (23-92)			6,5 (5-8)		
Grado de escolaridad	Primaria incompleta	106	61 (30-92)	0,399	0,007	4 (2-6)	0,854	0,001
	Primaria completa - secundaria incompleta	140	62 (31-122)			4 (2-6)		
	Más que secundaria	23	61 (30-92)			4 (2-6)		
Ocupación	Sin trabajo	122	61 (31-123)	0,335	0,007	4 (2-7)	0,232	0,009
	Otras ocupaciones	171	61 (30-95)			4 (2-6)		
	Ama de casa	38	82 (31-123)			4 (2-5)		
Condición del paciente	Nuevo	368	61 (31-122)	0,263	0,007	4 (2-6)	0,229	0,007
	Recaída	28	61 (31-94)			5 (3-9,5)		
	Abandono/recuperado	2	24 (17-31)			8,5 (4-13)		
Diagnóstico por rayos x de tórax	No	342	61 (31-120)	0,704	-0,019	4 (2-6)	0,004	-0,145
	Sí	55	61 (31-123)			3 (1-5)		
Diabetes mellitus	No	266	61 (30-105)	0,867	-0,009	4 (2-6)	0,095	-0,088
	Sí	94	61 (30-106)			4 (2-5)		
Hipertensión arterial sistémica	No	313	61 (31-95)	0,483	-0,038	4 (2-6)	0,786	-0,015
	Sí	32	46 (30-122)			4 (2,5-5)		

Para las variables dicotómicas se utilizó la prueba U de Mann-Whitney y, para las politómicas, la de Kruskal-Wallis.

‡ Rango intercuartílico; r = coeficiente de correlación de Pearson; r=Z/√N; E2R =épsilon al cuadrado; E2R = H/(n2-1) /(n+1)

La mediana del tiempo desde el inicio de síntomas hasta el diagnóstico confirmado de la enfermedad fue de 61 días (RIC: 31-120); la del tiempo desde la indicación de la baciloscopia hasta el inicio de tratamiento fue de 4 días (RIC: 2-6), y la del tiempo desde la confirmación del diagnóstico hasta el inicio del tratamiento fue de 0 días (RIC: 0-0). No hubo diferencias estadísticamente significativas en las medianas de tiempo entre el inicio de síntomas respiratorios y la confirmación del diagnóstico y las características sociodemográficas y clínicas de los casos (cuadros 2). Se encontró que la mediana de tiempo entre la indicación de la baciloscopia y el inicio del tratamiento fue menor en personas mayores de 65 años, con 3,5 días (RIC: 1-6; p*=*0,001); en los pacientes que además tuvieron radiografía de tórax indicativa de tuberculosis, la mediana fue de 3 días (RIC: 1-5; p*=*0,004), en tanto que fue mayor en las personas de otro tipo de población, con 6,5 días (RIC: 5-8; p*=*0,033) (cuadro 2).

En cuanto al resultado de diagnóstico oportuno, se incluyeron 398 registros que contaban con la información completa. De estos, 101 (25,4 %) de los casos tuvieron un diagnóstico oportuno, en comparación con 297 (74,6 %) casos que no lo tuvieron. Los pacientes que no tuvieron un diagnóstico oportuno eran, en su mayoría, hombres, con 62,3 % (185 casos); en el grupo de adultos se registraron 240 (80,8 %) casos; los participantes con primaria completa o secundaria incompleta eran 108 (53,7 %) y quienes tenían más que secundaria eran 15 (7,55 %); el 38 % (95 casos) no tenía trabajo, en tanto que el 49,2 % (123 casos) tenía alguna ocupación. En los casos con presencia de enfermedades crónicas, se encontró que el 25,9 % (69 casos) tenía diabetes mellitus y, el 7,9 % (20 casos), hipertensión arterial sistémica. Al calcular las razones de prevalencia, se encontró que estas no eran significativas, dado que se acercaban al valor nulo, con intervalos de confianza que incluían el 1 y valores de p no significativos (cuadro 3). Por último, se pudo evidenciar que las personas del grupo de adultos tuvieron mayor probabilidad de ser atendidos y diagnosticados a tiempo (HR ajustado=1.304; IC_95%_ 1,01- 1,69; p=0,043), en comparación con aquellos casos que se encontraban en la tercera edad (cuadro 4).

**Cuadro 3:** Oportunidad del diagnóstico de tuberculosis pulmonar por baciloscopia positiva según las características sociodemográficas y clínicas de los pacientes en dos establecimientos de salud de primer nivel de atención, Honduras, 2015-2019

Características		Diagnóstico oportuno (0-30 días) n=101		Diagnóstico no oportuno (>31 días) n=297		Total n=398		RP (IC_95%_)	p	RP ajustado (IC_95%_)	p
		n	%	n	%	n	%				
Sexo											
	Mujer	33	32,7	112	37,7	145	36,43	1	0,33	1	
	Hombre	68	67,3	185	62,3	253	63,57	0,95 (0,84-1,06)		0,86 (0,72-1,04)	0,12
Grupos de edad											
	Adultos	79	78,2	240	80,8	319	80,15	1		1	
	Tercera edad	22	21,8	57	19,2	79	19,85	1,04 (0,90-1,21)	0,59	0,99 (0,84-1,15)	0,85
Grado de escolaridad											
	Primaria incompleta	28	41,2	78	38,8	106	39,41	1		1	
	Primaria completa - secundaria incompleta	32	47,1	108	53,7	140	52,04	1,05 (0,91-1,21)	0,52	0,92 (0,81-1,05)	0,21
	Más que secundaria	8	11,8	15	7,5	23	8,55	0,89 (0,64-1,22)	0,46	0,81 (0,55-1,18)	0,27
Ocupación											
	Sin trabajo	27	33,3	95	38,0	122	36,86	1		1	
	Todas las ocupaciones	48	59,3	123	49,2	171	51,66	0,92 (0,81-1,06)	0,24	0,95 (0,80-1,14)	0,6
	Ama de casa	6	7,4	32	12,8	38	11,48	1,08 (0,92-1,28)	0,36	1,10 (0,91-1,34)	0,31
Grupo de riesgo											
	Ninguno	77	85,6	232	86,6	309	86,31	1		1	
	Alguno	13	14,4	36	13,4	49	13,69	0,98 (0,82-1,17)	0,81	1,07 (0,82-1,39)	0,61
Diabetes mellitus											
	No	69	73,4	197	74,1	266	73,89	1		1	
	Sí										
Hipertensión arterial sistémica											
	No	25 79	26,6 86,8	69 234	25,9 92,1	94 313	26,11 90,72	0,99 (0,86-1,14) 1	0,90	1,12 (0,87-1,44) 1	0,36
	Sí	12	13,2	20	7,9	32	9,28	0,84 (0,63-1,10)	0,20	0,85 (0,59-1,22)	0,38
Otras enfermedades											
	No	74	84,1	215	85,3	289	85,00	1		1	
	Sí	14	15,9	37	14,7	51	15,00	0,94 (0,82-1,07)	0,33	0,92 (0,72-1,18)	0,53

**Cuadro 4:** Regresión de Cox de los casos de tuberculosis pulmonar con baciloscopia positiva, por edad, sexo y diagnóstico de rayos x en dos establecimientos de salud de primer nivel de atención, Honduras, 2015-2019

Covariable		n (%)	HR crudo (IC_95%_)	p	HR ajustado (IC_95%_)	p
Edad						
	Tercera edad	79 (19,8)	1		1	
	Adultos	319 (80,2)	1,336 (1,04-1,72)	0,023	1,304 (1,01- 1,69)	0,043
Diagnóstico por rayos X						
	No	342 (86,1)	1		1	
	Sí	55 (13,9)	0,7775 (0,58-1,04)	0,091	0,834 (0,619-1,12)	0,233
Sexo						
	Mujer	145 (36,4)	1			
	Hombre	253 (63,6)	1,163 (0,95-1,43)	0,152		

En los cuadros 5 y 6 se presentan las barreras y los elementos facilitadores del diagnóstico y el tratamiento oportuno de la tuberculosis según la experiencia del personal de salud, los pacientes y los familiares. A continuación, se presentan en forma textual algunos relatos de los pacientes y del personal de salud.

**Cuadro 5:** Barreras y elementos facilitadores para el diagnóstico y tratamiento oportuno de la tuberculosis según el personal de salud

Nivel de modelo ecológico-social (18-20)	Barreras	Elementos facilitadores
Intrapersonal	Precarias condiciones socioeconómicas del paciente Automedicación del paciente antes de recibir el diagnóstico Paciente acude tarde a consultar en los establecimientos de salud	Idea de tener conocimiento de la enfermedad y disposición de los protocolos en los establecimientos de salud Experiencia satisfactoria del personal de salud que labora en el Programa Nacional de Tuberculosis (PNT)
Interpersonal	No hay personal de psicología que apoye al paciente durante el diagnóstico y el tratamiento de la enfermedad. Pérdida de incentivos económicos para el paciente en el proceso de tratamiento	Apoyo y consejería brindada por el personal de salud al paciente con tuberculosis, su autocuidado y los cuidados con los convivientes
Sistema de salud	Rotación continua del personal de enfermería asignado al PNT Recibir otros diagnósticos previos a la enfermedad en clínicas privadas Falta de laboratorio para procesar muestras Falta de transporte para realizar búsquedas activas Espacio físico de consulta inadecuado	Control supervisado de los casos Disponibilidad del tratamiento que permite su pronto inicio Reserva del tratamiento de la tuberculosis
Social y comunitario	Límites fronterizos impuestos por maras y pandillas El estigma social del paciente por causa de la enfermedad	Las organizaciones exteriores y su apoyo
Político-administrativo	Hay pasos adicionales en la entrega del medicamento El funcionamiento horizontal del sistema de salud involucra mucha gente.	El funcionamiento horizontal no impide seguir asumiendo el PNT como programa.

**Cuadro 6:** Barreras y elementos facilitadores para el diagnóstico y tratamiento oportuno de la tuberculosis según pacientes y familiares

Nivel de modelo ecológico-social (18-20)	Barreras	Elementos facilitadores
Intrapersonal	Creencias sobre la enfermedad que no corresponden a la información científica	Idea de tener el conocimiento de la enfermedad
Interpersonal	No se señalaron	Apoyo familiar y de grupos sociales
Sistema de salud	Desarticulación del sistema de salud público y el privado El personal de salud pasa por alto la sintomatología de la enfermedad. Gastos por tratamientos equivocados previos al diagnóstico de la tuberculosis Efectos adversos del tratamiento que afectan la salud del paciente Estructura física del establecimiento de salud no tiene las condiciones para personas con capacidades especiales.	Buena atención y actitud de servicio por parte del personal de salud Personal de salud atento a brindar el tratamiento de tuberculosis Explicación adecuada de la enfermedad por parte del personal de salud Disponibilidad del tratamiento en el establecimiento de salud, lo que permite su inicio oportuno
Social y comunitario	El estigma social de los pacientes a causa de laenfermedad Límites fronterizos entre maras y pandillas	La comunicación y su influencia en el individuo

*“*Y otra cosa es la economía de los pacientes, en la mayoría de los casos, los pacientes que tienen tuberculosis o no trabajan o tienen un trabajo, como de botar basura con la municipalidad, de transportista, ayudante de bus*”.* (PS6: condiciones socioeconómicas precarias como barreras del paciente con tuberculosis).

*“*Y la otra era que se van a las clínicas privadas y es donde de repente dan otros diagnósticos.*”*(PS1: falta de articulación del sistema de salud público y el privado como barrera).

“… pero a mí me atendieron excelente y creo que no es por eso, he visto a los demás pacientes que vienen también en ese momento ellas son bien atentas con ellos, están pendientes que se tomen el medicamento, le entregan, le dan el agua a uno y todo”. (PT5: elemento facilitador por la buena atención y actitud de servicio del personal de salud).

“Cuando a mí me empezó yo buscaba médicos privados generales que me ponían medicamentos y me calmaba, pero realmente no sabían qué era lo que tenía, cuando me vinieron a detectar tuberculosis ya, ya había perdido 17 libras de peso, estaba agotado físicamente.” (PT6: falta de articulación del sistema de salud público y el privado como barrera).

“Y entonces, incluso una vez que lo llevamos a él, había unos muchachos que nos querían bajar del carro, entonces ya por eso nosotros decidimos no llevarlo a él”. (FL7: límites fronterizos entre maras y pandillas como barrera).

Con el modelo descrito se logró una mayor comprensión de las categorías en los distintos niveles, y de los diferentes relatos y experiencias de los entrevistados.

**Figura 1:**
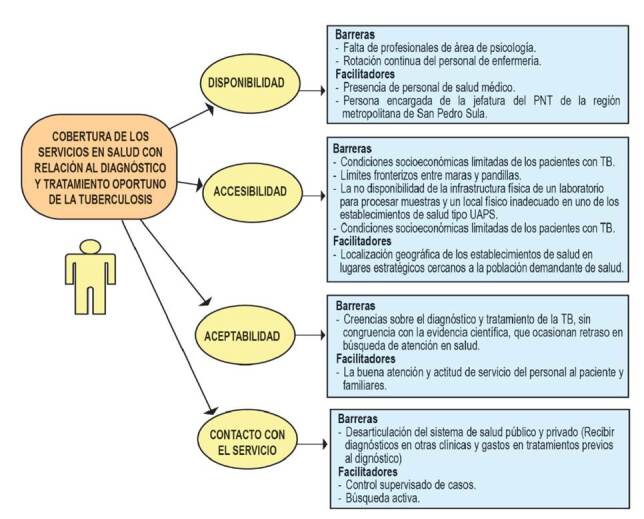
Mapa conceptual de la descripción de las barreras y elementos facilitadores que afectan la cobertura del Programa Nacional de Tuberculosis en San Pedro Sula, Honduras

En la figura 1, se presenta un mapa conceptual explicativo según el modelo de cobertura de Tanahashi, que permite comprender cómo las diferentes barreras y elementos facilitadores relatados por los entrevistados afectaron la cobertura del Programa Nacional de Tuberculosis ^23^.

## Discusión

En este estudio se determinaron, describieron y analizaron las barreras y los elementos facilitadores del diagnóstico y el tratamiento que afectan la cobertura del programa de tuberculosis en dos instituciones de salud de primer nivel de San Pedro Sula, Honduras. Se encontró que la mayoría de los pacientes con diagnóstico de tuberculosis era de sexo masculino, la mitad tenía 43 años o más, eran mestizos y con un nivel de escolaridad bajo; la tercera parte de ellos no tenía trabajo y quienes sí lo tenían estaban en el sector informal de la economía. Todo ello concuerda con lo hallado en otros estudios de poblaciones con características sociodemográficas y económicas similares y, en general, se destaca la precariedad socioeconómica de los pacientes con la enfermedad ^24^^-^^26^.

La mediana de días entre el inicio de síntomas respiratorios y la confirmación del diagnóstico de tuberculosis, fue de 61 días, semejante a los resultados en países como Brasil y España, en donde el retraso del diagnóstico osciló entre 60 y 61 días ^27^^,^^28^. En cuanto a los días entre la indicación de la baciloscopia y el comienzo del tratamiento, la mediana fue de 4 días, lo que se contrapone a lo encontrado en el estudio de Rodríguez, *et al.*, con una mediana de 6 días en su población de estudio ^29^, probablemente debido a que dicho periodo depende principalmente del sistema de salud y no del paciente.

De aquellos pacientes con retraso en el diagnóstico, el 25,9 % tenía diabetes mellitus y el 7,9% sufría hipertensión arterial sistémica. En el 2018, en un estudio de Htun en el sudeste asiático, se encontraron retrasos prolongados en el diagnóstico de los pacientes con tuberculosis y diabetes mellitus, lo cual se asoció con la doble carga de estas enfermedades y el escaso conocimiento de los síntomas de la tuberculosis ^30^. En el estudio de Gaviria, *et al.*, en Colombia en el 2010, se mencionó como posible causa del retraso en el diagnóstico de la tuberculosis el tener hipertensión arterial u otras enfermedades, ya que su presencia pudo haber “distraído” al médico ^31^.

Según lo referido por los entrevistados, las principales barreras para el diagnóstico fueron, en primer lugar, las precarias condiciones socioeconómicas de los pacientes, lo que coincide con un estudio de Getnet, *et al.,* en el 2019, en el que se menciona que las características relacionadas con los factores socioeconómicos y demográficos influyeron en la búsqueda de servicios de salud ^32^.

Con respecto a los pacientes de nuestro estudio con retraso del diagnóstico de tuberculosis, el 49,2 % pertenecía al sector informal de la economía y el 38 % no tenía trabajo. En segundo lugar, los aspectos culturales y creencias de los pacientes sobre la enfermedad en ocasiones se oponen a la evidencia científica. Entre los pacientes de nuestro estudio con demora en el diagnóstico, el 53,7 % tenía un nivel de escolaridad de primaria incompleta o secundaria incompleta y solo el 7,5 % había superado la secundaria. En tercer lugar, los límites fronterizos impuestos por los grupos delictivos organizados (maras y pandillas) fueron mencionados por los entrevistados, pues a raíz de esta situación se les dificultaba asistir a los establecimientos de salud. En el departamento de Cortés, la mayoría de los grupos delictivos organizados se concentra en una zona geográfica determinada de San Pedro Sula ^33^. En cuarto lugar, la desarticulación entre los sectores público y privado del sistema de salud fue frecuentemente señalada por los entrevistados; en este sentido, los pacientes informaron haber asistido a clínicas privadas donde en ocasiones no obtuvieron el diagnóstico ni el manejo correcto de la tuberculosis. En un estudio del 2017 de Paramasivam, *et al.*, en Kerala, se estableció que los proveedores privados de servicios de salud no tenían vínculos fuertes con el sistema de salud del gobierno y que el retraso en el diagnóstico de tuberculosis fue mayor en quienes acudieron a instituciones privadas que en los servicios gubernamentales ^34^. En quinto lugar, tras las reformas del sistema de salud de Honduras, el funcionamiento horizontal de la SESAL y su manejo del sistema de vigilancia de la tuberculosis (antes constituido como Programa Nacional de Tuberculosis), probablemente no ha contribuido a mejorar la problemática de la tuberculosis. En Colombia, según los estudios de Arbeláez, *et al.*, en el 2006 y de Ayala en el 2002, las reformas en el sector de la salud relacionadas con la descentralización y la pérdida de la verticalidad de los programas no han favorecido la situación epidemiológica de la tuberculosis ^35^^,^^36^.

En cuanto a los factores facilitadores, los entrevistados expresaron que la buena atención y la actitud del personal de salud contribuyeron a su diagnóstico y tratamiento oportunos. Según el estudio de Gebremariam, *et al.*, la mayoría de los pacientes se sintieron contentos con la forma en que los profesionales de la salud los recibían y trataban en los centros de salud. Algunos dijeron que ello los incentivó para recibir el tratamiento, pues los profesionales de la salud los recibían con una “buena cara” y los alentaban a terminarlo ^37^.

Con respecto a las diferentes barreras del tratamiento de la tuberculosis, los entrevistados refirieron que los gastos en tratamientos previos representaban un alto costo. Según el estudio de Awoke, *et al.*, en el sur de Etiopia en el 2017, el tratamiento previo inespecífico antes del diagnóstico de la enfermedad se estableció como un factor asociado con el retraso en el tratamiento de la tuberculosis ^38^.

En nuestro estudio, la mayoría de los pacientes recibió un tratamiento oportuno, resultado igual al reportado en el estudio de Peri, *et al.*^39^. Según lo narrado por los entrevistados, algunos de los elementos facilitadores fueron la disponibilidad y la reserva del tratamiento en los establecimientos de salud.

Entre las fortalezas del estudio, cabe mencionar su ejecución en el municipio de San Pedro Sula, lugar en donde la tuberculosis ha sido un problema de importancia, así como la detección de las barreras y los elementos facilitadores del diagnóstico y el tratamiento de la tuberculosis desde la visión amplia del personal de salud, los pacientes y sus familiares. Se pudo determinar que algunos de los puntos de los pilares 1 y 2 de la estrategia “Fin de la TB” no se han implementado en su totalidad. Entre las limitaciones del estudio, debe mencionarse la calidad de las fuentes secundarias, pues no se contó con la fecha exacta de inicio de los síntomas respiratorios del paciente y otras fechas de interés, lo que pudo afectar el tamaño de la muestra y, con ello, la posibilidad de encontrar diferencias significativas.

Por último, se encontró que en la mayoría de los casos no hubo un diagnóstico oportuno de la tuberculosis como resultado de algunas de las barreras referidas por el personal de salud, los pacientes y sus familiares, lo que ha afectado la cobertura del Programa Nacional de Tuberculosis en San Pedro Sula (cuadro 6).

Con base en estos hallazgos, se sugiere que la SESAL coordine con otras instancias gubernamentales el suministro de apoyo económico a los pacientes con tuberculosis en situación de pobreza, y coordine equipos de trabajo intersectoriales en aquellos sitios con límites fronterizos impuestos por maras y pandillas. Se propone la implementación de charlas educativas en clubes para pacientes y sus familias, y la difusión de mensajes educativos sobre la enfermedad por medio de la radio, la televisión y el sistema educativo formal. Asimismo, debe darse prioridad al enlace entre la SESAL y el sistema de salud privado por medio de protocolos estrictos de evaluación de los pacientes con sospecha de tuberculosis. Se recomienda la creación de sistemas digitales de rápido acceso para la clínica de la enfermedad en los establecimientos de salud, de manera que pueda obtenerse una información más completa del paciente. Todo ello contribuiría a fortalecer la vigilancia epidemiológica de la tuberculosis en Honduras dada la ausencia de un programa nacional de prevención y control como tal.
